# Systematic review of prognostic factors for work participation in patients with sciatica

**DOI:** 10.1136/oemed-2019-105797

**Published:** 2019-07-11

**Authors:** Teddy Oosterhuis, Veerle R Smaardijk, P Paul FM Kuijer, Miranda W Langendam, Monique H W Frings-Dresen, Jan L Hoving

**Affiliations:** 1 Coronel Institute of Occupational Health, Amsterdam UMC, University of Amsterdam, Amsterdam Public Health Research Institute, Amsterdam, The Netherlands; 2 Department of Clinical Epidemiology, Biostatistics and Bioinformatics, Amsterdam UMC, University of Amsterdam, Amsterdam Public Health Research Institute, Amsterdam, The Netherlands

**Keywords:** epidemiology, musculoskeletal

## Abstract

Sciatica impacts on the ability to work and may lead to a reduced return to work. This study reviewed and summarised prognostic factors of work participation in patients who received conservative or surgical treatment for clinically diagnosed sciatica. We searched MEDLINE, CINAHL, EMBASE and PsycINFO until January 2018. Cohort studies, using a measure of work participation as outcome, were included. Two independent reviewers performed study inclusion and used the Quality In Prognosis Studies tool for risk of bias assessment and GRADE to rate the quality of the evidence. Based on seven studies describing six cohorts (n=1408 patients) that assessed 21 potential prognostic factors, favourable factors for return to work (follow-up ranging from 3 months to 10 years) included younger age, better general health, less low back pain or sciatica bothersomeness, better physical function, negative straight leg raise-test, physician expecting surgery to be beneficial, better pain coping, less depression and mental stress, less fear of movement and low physical work load. Study results could not be pooled. Using GRADE, the quality of the evidence ranged from moderate to very low, with downgrading mainly for a high risk of bias and imprecision. Several prognostic factors like pain, disability and psychological factors were identified and reviewed, and these could be targeted using additional interventions to optimise return to work. PROSPERO registration number: CRD42016042497.

**Key messages**

**What is already known about this subject?**

- Sciatica impacts on the ability to work and may lead to a reduced return to work.
- Prognostication is important for physicians but guidance in the prognostication process regarding return to work in patients with sciatica is lacking.

**What are the new findings?**

- Physicians can assess whether a worker is more likely to return to work by assessing prognostic factors. Favourable factors for return to work include younger age, better general health, less low back pain or sciatica bothersomeness, better physical function, negative SLR-test, physician expecting surgery to be beneficial, better pain coping, less depression and mental stress, less fear of movement and low physical work load.

**How might this impact on policy or clinical practice in the foreseeable future?**

- Prognostic factors like pain, disability and psychological factors can be used in the prognostication process. More importantly, these prognostic factors can be targeted by referring for additional interventions in order to promote return to work.

## Introduction

Lumbosacral radicular syndrome, often called sciatica, is commonly caused by a herniated lumbar disc.^1^ The syndrome is characterised by lower limb pain radiating below the knee in an area of the leg served by one or more lumbosacral nerve roots. There may be other neurological findings such as sensory and motor deficits. Sciatica is usually self-limiting with pain and disability decreasing over time,^2^ but not all patients fully recover.^2–4^ Surgical treatment is usually offered in more severe cases when severe radiating leg pain persists after a period of conservative management.^5^ In a large study (n=782), 34% of conservatively treated patients experienced very or extremely bothersome symptoms at 6 months follow-up.^3^ Similarly, a systematic review (n=13 883) showed that surgically treated patients reported, despite decreased pain and disability scores 3 months after surgery, on average mild to moderate pain and disability 5 years after surgery.^4^

The direct and indirect costs of patients suffering from sciatica are high,^6^ and an important cost driver is work absenteeism.^7–9^ In the acute phase, most people with sciatica will stop working and some will resume work in the short time. Return to work (RTW) rates vary from 66% after 2 years^10^ to between 67% and 85% after 10 years.^11^ The high socioeconomic impact of sciatica and its impact on the ability to work in patients raise the need to identify factors that predict reduced RTW. Prognostic evidence could assist clinicians to better define high risk groups and inform both clinicians and patients with regard to counselling and treatment choices to promote RTW. The objective of this study was to review and summarise prognostic factors of work participation in patients with sciatica.

## Methods

This review is reported according to the Preferred Reporting Items for Systematic Reviews and Meta-Analyses guidelines.^12^

### Eligibility criteria

We included full-text original articles of studies concerning adults (≥18 years) clinically diagnosed with sciatica, who received either conservative treatment or surgical treatment. Studies with participants having stenosis or cauda equina syndrome were excluded, if no separate data were available for participants without stenosis or cauda equina syndrome. We included cohort studies that evaluated any possible prognostic factor associated with RTW as a measure of work participation.

### Search

We searched relevant cohort studies using MEDLINE via PubMed, CINAHL via EBSCOhost, EMBASE and PsycINFO via OVID, from inception until January 2018. Specific search terms for the population, work participation and prognostic filters were used. The search strategy was developed with input from the review team and a clinical librarian, based on search strategies for sciatica using the search strategy for the 2016 NICE guideline on lumbosacral radicular syndrome,^13^ the published MEDLINE filter for prognostic studies^14^ and Yale University’s methodological research filter for prognosis and natural history.^15^ The clinical librarian developed the string for work participation. The search strategy was adapted for each database. Furthermore, references in relevant reviews and in identified cohort studies were screened. We did not apply any language restrictions. Online supplementary appendix 1 shows the search strategy used in MEDLINE.

10.1136/oemed-2019-105797.supp1 Supplementary file 1

### Study selection

Pairs of review authors (TO, VRS, PK, MHWFD and JLH) independently selected the studies to be included by applying the selection criteria. First, title and abstract screening was performed using Covidence (covidence.org). Subsequently, full-text articles of potentially relevant studies were retrieved and assessed. Disagreements were resolved using consensus.

### Data collection process

Using a standardised form, one reviewer (TO or VRS) extracted data from the included studies. A second reviewer (TO, VRS, PK or JLH) checked the results. Data that were extracted included first author, year, country; case definition; source population; characteristics of the study population; inclusion and exclusion criteria; sample size, including number of complete cases; prognostic factors and potential confounders including their measurement method; definition of RTW as the work participation outcome; description of the content of treatment (eg, surgery, rehabilitation and other conservative); length of follow-up; analysis used (univariable or multivariable regression); extracted or calculated ORs or HRs with 95% CIs, if sufficient data were available and source of funding. Study authors were contacted in case of insufficient information on any of these items. In case of multiple follow-up moments per study, the latest follow-up was used.

### Risk of bias in individual studies

Pairs of review authors (TO, VRS, PK, MWL and JLH) independently assessed the risk of bias of the included studies by using the Quality In Prognosis Studies (QUIPS) tool.^16^ At the study level, six domains were rated as high, moderate or low risk of bias according to the QUIPS guidelines. Finally, an overall risk of bias was determined per study: low, moderate or high risk. Consensus was used to resolve disagreements. If no agreement was reached, a third reviewer was consulted. Study authors were contacted in case of insufficient information to assess the risk of bias.

### Synthesis of results

Meta-analyses were planned with a random-effects meta-analysis model, but only if populations, prognostic factors, outcomes and time points were sufficiently homogeneous across studies.^17^ We considered RTW outcomes of 6 months or more as a long-term follow-up. We planned separate analyses for (1) studies reporting ORs and HRs, (2) studies assessing surgical and non-surgical populations and (3) different non-surgical approaches. If meta-analyses were not feasible, we performed a narrative synthesis.^18^

The Grading of Recommendations Assessment, Development and Evaluation (GRADE) approach was used to assess the overall quality of the evidence.^19^ Evidence from explorative cohort studies started as moderate quality evidence. Evidence from confirmative cohort studies started as high-quality evidence.^20^ The quality of the evidence was downgraded according to the performance of the studies against five domains: risk of bias (<75% of participants from studies with a low risk of bias), inconsistency (point estimates of both OR >1.0 and OR <1.0 in meta-analyses; no overlap in CIs in meta-analyses), indirectness and imprecision (fewer than 10 participants per prognostic factor or category in case of categorical variables; non-significant results; CIs crossing OR=0.5 or 2.0^21^; fewer than 100 cases reaching endpoint). Publication bias was assessed through the construction of funnel plots only if 10 studies or more were included in the meta-analyses.

## Results

**Figure 1:**
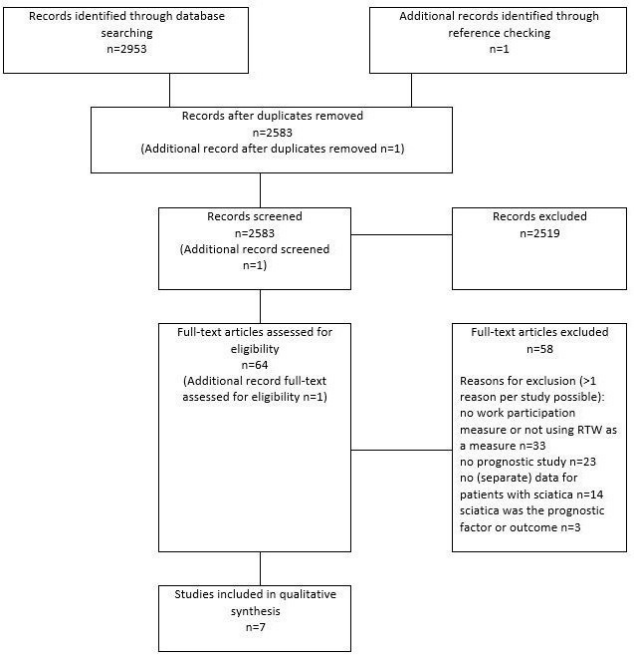
Flow diagram. RTW, return to work.

The search yielded 2953 articles: MEDLINE 627, CINAHL 853, EMBASE 1396 and PsycINFO 77. After removal of duplicates, 2583 articles remained (figure 1). After screening titles and abstracts, 64 full-texts were read. Of these, six articles (that described five unique cohorts) fulfilled all eligibility criteria. Screening of reference lists of included studies identified one more eligible study.^22^ This resulted in seven studies included in total. Reasons for exclusion of 58 full-texts were (>1 reason per study possible): no work participation measure or not using RTW as a work participation measure (33), no prognostic study (23), no data or no separate data for patients with sciatica (14) and sciatica was the prognostic factor or outcome (3). Online supplementary appendix 2 provides an overview of all excluded studies.

### Study characteristics

Table 1 shows the characteristics of the seven included studies describing six cohorts. All 1408 patients were diagnosed with a lumbar disc herniation. Three publications, describing two cohorts,^23–25^ included a mixed population consisting of both conservatively and surgically treated patients. In these cohorts, 30%^25^ and 53%^23^ of the patients received surgery, at 2 and 4 years follow-up, respectively. One of these studies controlled the analysis for initial type of treatment.^24^ The four remaining studies included surgical patients only^22 26–28^ including one that consisted of patients with reoperation for recurrent herniation.^27^ Three cohorts were from North-America and three were from Europe. The number of participants varied from 46 to 394 per cohort, mean age ranged from 35 to 46 years, 28%–78% were male and all analyses included working populations. Two cohorts measured short-term follow-up at 3^28^ and 6 months.^26^ Long-term outcomes ranged from 2,^22 25^ 3,^27^ 4^23^ to 10 years^24^ follow-up, with the majority measured between 2 and 4 years. All studies used self-reported RTW which was measured in various ways: being employed,^23 24 28^ return to usual number of work hours per week,^26^ return to full-time work,^25^ return to ‘any’ work,^22^ the ability to work at least 6 months.^27^ All studies used multiple regression analysis (adjusted ORs) and all but one reported ORs. This one study^22^ reported betas (ln), which we converted to ORs. Clinical heterogeneity, differences in RTW measures and the use of different sets and measurements of prognostic factors, confounders and follow-up time points precluded pooling of data or performance of any subgroup analyses given the limited number of studies.

**Table 1:** Study characteristics of the seven included studies

Author, year, country	Population	Inclusion and exclusion criteria	Sample size, complete cases	Prognostic factors and confounders	Outcome, time point, case definition	Analysis	Results
Atlas *et al*, 2000, USA^23^	Working patients with diagnosed disc herniation, who received conservative or surgical (n=174/327; 53%) treatment, 68% male, mean age 40 years	Inclusion: diagnosed lumbar disc herniation Exclusion: previous spine surgery, cauda equina, spinal or other comorbidity, pregnancy	n=440, n=404 completed at least one follow-up, n=327 completed last follow-up	Receiving workers’ compensation, level of education, duration current episode, comorbidity, age, SF-36 general health, low back pain	Return to work; 4-year follow-up; case definition working on a job for pay	Stepwise multiple logistic regression	Receiving workers’ compensation OR 0.6 (0.3 to 1.2); age OR 0.7 (0.6 to 0.8); general health OR 1.1 (1.0 to 1.2); low back pain OR 0.8 (0.6 to 0.9), model adjusted for the other variables
Atlas *et al, 2006*, USA^24^	Working patients with sciatica, who received conservative or surgical treatment, 66% male, mean age 40 years	Inclusion: diagnosed lumbar disc herniation Exclusion: cauda equina, spinal or other comorbidity, pregnancy	n=394, who completed at least one follow-up between 5 and 10 years, n=352 competed last follow-up	Age, gender, initial treatment, physician expectation of surgery benefit, Quebec classification, category 4/6, low back frequency score, SF-36 physical function, SF-36 mental health	Return to work; 10 years; case definition employed at follow-up	Stepwise multiple logistic regression	Age OR 0.42 (0.3 to 0.58), male gender OR 0.33 (0.09 to 1.0), initial treatment, surgical OR 1.4 (0.46 to 4.6), Physician expected benefit OR 5.0 (1.65 to 17.7), physical function OR 1.4 (1.1 to 1.8), Quebec classification NS, Low Back Frequency Score NS, mental health NS, model adjusted for independent baseline prognostic factors
den Boer *et al*, 2006, Netherlands^26^	Working patients who underwent lumbar disc surgery, 59% male, mean age 41 years	Inclusion: >18 years, failure of conservative treatment, understand and read Dutch, having a paid job before the episode of complaints started. Exclusion: previous back surgery, physical comorbidity	n=200, n=182 complete cases	Fear of movement/ (re)injury, passive pain coping, physical work load, job satisfaction, duration sick leave Confounders: education, disability presurgery, neurological deficits presurgery, pain 3 days postsurgery	Work capacity, 6 months postsurgery, case definition: percentage work capacity (hours/week) compared with work capacity before the pain episode started=100%	Multiple logistic regression, only variables significant in univariable regression were entered into the model, and prespecified confounders	Fear of movement/ (re)injury OR 1.09 (SE 0.04 calculated 95% CI 1.01 to 1.18), passive pain coping OR 1.08 (SE 0.04, calculated 95% CI 1.0 to 1.17), physical work load OR 1.19 (SE 0.06, calculated 95% CI 1.06 to 1.34), job satisfaction OR 0.98 NS, duration sick leave OR 1.26 NS
Grøvle *et al, 2013*, Norway^25^	Working patients with sciatica and disc herniation, who underwent conservative or surgical (30%) treatment, 69% male, mean age 44 years	Inclusion:≥18 years, radiating pain below the knee and/or paresis, lumbar disc herniation Exclusion: pregnancy, spinal fracture, tumour, infection, previous surgery same disc, not able to read Norwegian	n=297, n=237 included complete cases (n=9 who were student, retired or homemaker at follow-up, were excluded)	Age, gender, marital status, current smoker, duration current sciatica episode > 3 months, had sciatica before, duration back problems>1 year, subjective health complaints, sciatica bothersomeness, disability, fear avoidance beliefs (work), fear of movement/reinjury, general health, emotional distress back pain, leg pain, positive SLR, motor weakness, reflexes depressed, sensory decrease	Return to work, 2 years; case definition: return to full-time work by self-report	Multiple logistic regression, only variables significant in univariable regression were entered into the model (p values < 0.2)	Age OR 0.97 (0.93 to 1.00), female OR 0.61 (0.31 to 1.22), bothersomeness OR 0.89 (0.82 to 0.97), fear avoidance beliefs OR 0.93 (0.90 to 0.97), general health OR 1.03 (1.01 to 1.05), positive SLR OR 0.44 (0.20 to 0.95)
O’Donnell *et al, 2017, USA* 27	Workers’ compensation patients who received reoperation discectomy with or without fusion, 77.2% male, mean age 39.4 years	Inclusion: lumbar disc herniation after workplace injury, receiving lost-work compensation, injuries between 2005 and 2012, same level revision surgery exclusion: spondylolisthesis, spinal deformity, vertebral fractures, epidural haematomas and abscesses, spinal tumours, smoking history or using smoking deterrents	n=298, n=196 with fusion, retrospective cohort, therefore only complete cases	Revision surgery: fusion or no fusion, age, sex, marital status, labor-intensive occupation, permanent disability benefits, legal representation, psychiatric comorbidities, physical therapy and chiropractic care, opioid analgesic use, household income, permanent disability, time from primary surgery to reoperation surgery	Return to work, 3 years; case definition: ability to return within 2 years and work for at least 6 months within 3 years	Multiple logistic regression	Revision surgery: fusion OR 0.56 (0.33 to 0.97), psychiatric comorbidity before revision surgery OR 0.19 (0.05 to 0.68), opioids use within 1 month of revision surgery OR 0.44 (0.26 to 0.75)
Schade *et al*, 1999, Switzerland^22^	Patients who underwent lumbar disc surgery, 74% male, mean age 35 years (demographic data from Boos *et al*)^38^	Inclusion: a scheduled discectomy, age 20–50 years, continued employment at the time of surgery, no previous back surgery, failed conservative treatment, availability for additional clinical+MRI examination Exclusion: no Swiss residency, rapid progressive severe motor deficit or cauda equina syndrome	n=46, n=42 complete cases	Anxiety, depression, self-control, well-being, vitality, general health, occupational mental stress, job satisfaction, job-related resignation, social support confounders: pain and/or disability presurgery	Return to work, 2 years; case definition: return to ‘any’ work (time in months)	Univariable regression and stepwise multiple regression (medical data, general psychological factors and psychosocial aspects of work)	Depression beta ln 0.43 (estimated OR 1.54), occupational mental stress beta ln 0.28 (estimated OR 1.32), pain and/or disability presurgery beta ln 0.35 (estimated OR 1.42)
Than *et al, 2016, USA* 28	Patients who underwent lumbar discectomy, 51% male, mean age 45 years	Inclusion: 18–80 years, symptomatic lumbar disc herniation recalcitrant to non-invasive therapies for at least 6 weeks exclusion: history of previous lumbar spinal surgery at the level of disc herniation; significant motor weakness (such as foot drop) or cauda equina syndrome; cancer, infection or fracture involving any portion of the spine; pregnancy	n=127, n=123 complete cases at 1-year follow-up	Physical function/general health (SF-36 scale), physical function (Oswestry Disability Index), BMI, back pain (VAS), age, sex, insurance type, work status, smoking status, baseline health status measures, self-reported work/disability status	Return to work, 3 months, case definition: employed at 3 months following the lumbar discectomy	Stepwise logistic regression	Age OR 0.92 (0.85 to 0.99), male sex OR 0.22 (0.04 to 1.09), BMI OR 0.90 (0.78 to 1.04), general health OR 1.03 (0.98 to 1.08), physical function OR 1.06 (0.997 to 1.13), smoking status OR 4.37 (0.82 to 23.27)

OR with 95% CI in brackets.

### Risk of bias within studies

Table 2 shows the results of the risk of bias assessment, using QUIPS.^16^ Four studies had an overall low risk of bias,^23 25–27^ of which two studies scored a low risk of bias on all six domains.^25 26^ Three studies had an overall moderate risk of bias.^22 24 28^

**Table 2:** Risk of bias assessment of the seven included studies

Study	QUIPS domain 1	QUIPS domain 2	QUIPS domain 3	QUIPS domain 4	QUIPS domain 5	QUIPS domain 6	QUIPS overall score
Atlas *et al* 23	Low	Moderate	Low	Low	Low	Low	Low
Atlas *et al* 24	Low	High	Moderate	Moderate	Low	Moderate	Moderate
den Boer *et al* 26	Low	Low	Low	Low	Low	Low	Low
Grøvle *et al* 25	Low	Low	Low	Low	Low	Low	Low
O’Donnell *et al* 27	Low	Low	Low	Low	Low	Moderate	Low
Schade *et al* 22	Moderate	High	Moderate	Low	Moderate	Low	Moderate
Than *et al* 28	Low	Moderate	Moderate	Moderate	Moderate	Low	Moderate

QUIPS domain 1: The study sample adequately represents the population of interest.

QUIPS domain 2: The study data available (ie, participants not lost to follow-up) adequately represent the study sample.

QUIPS domain 3: The prognostic factor is measured in a similar way for all participants.

QUIPS domain 4: The outcome of interest is measured in a similar way for all participants.

QUIPS domain 5: Important confounders are appropriately accounted for.

QUIPS domain 6: The statistical analysis is appropriate, and all primary outcomes are reported.

### Prognostic factors

In total, 21 potential prognostic factors for RTW were assessed. All factors were derived from multiple regression models. Some factors were assessed at different time points. Age and sex were included in three studies, general health and fear avoidance beliefs were included in two studies. Four pain measures were used in four studies: back pain intensity, back pain frequency, sciatica bothersomeness and opioid use. The results of all studies are summarised below. All studies measured RTW, but reported prognostic factors for either RTW or reduced RTW. This lead to ORs both >1 and <1 for similar prognostic factors, despite all associations being in the same direction for the same factors.

#### RTW in both mixed and surgical populations

Workers with less fear avoidance beliefs were more likely to RTW at 6 months (OR 1.09 more fear avoidance - less RTW, SE 0.04, estimated 95% CI 1.01 to 1.18, surgical population)^26^ and 2 years (OR 0.93 less fear avoidance - more RTW; 95% CI 0.90 to 0.97, mixed population).^25^

#### RTW in mixed populations

Younger age did not predict RTW at 2 years (OR 0.97; 95% CI 0.93 to 1.00),^25^ but predicted RTW at 4 years (OR 0.7; 95% CI 0.60 to 0.80^23^) and 8 years (OR 0.42; 95% CI 0.30 to 0.58^24^). Better general health predicted RTW at 2 years (OR 1.03; 95% CI 1.01 to 1.05^25^) and 4 years (OR 1.10; 95% CI 1.00 to 1.20^23^). Less sciatica bothersomeness predicted RTW at 2 years (OR 0.89; 95% CI 0.82 to 0.97^25^), lower low back pain intensity predicted RTW at 4 years (OR 0.80; 95% CI 0.60 to 0.90^23^) and better physical function predicted RTW at 10 years (OR 1.40; 95% CI 1.10 to 1.80^24^). A positive SLR test predicted reduced RTW at 2 years (OR 0.44; 95% CI 0.20 to 0.95^25^). The physician expecting surgery to be beneficial predicted RTW at 10 years (OR 5.00; 95% CI 1.65 to 17.70^24^). No association with RTW was found for: sex (female OR 0.61; 95% CI 0.31 to 1.22^25^; male OR 0.33; 95% CI 0.09 to 1.00^24^), receiving workers’ compensation (OR 0.60; 95% CI 0.30 to 1.20^24^), initial surgical treatment (OR 1.40; 95% CI 0.46 to 4.60^24^), low back pain frequency (no data presented^24^), the Quebec classification (no data presented^24^) and mental health (no data presented^24^).

#### RTW in surgical populations

Older age (OR 0.92; 95% CI 0.85 to 0.99) predicted reduced RTW at 3 months.^28^ Passive pain coping (OR 1.08, SE 0.04, estimated 95% CI 1.00 to 1.17^26^ and higher physical work load (OR 1.19, SE 0.06, estimated 95% CI 1.06 to 1.34)^26^ predicted reduced RTW at 6 months. Depression (estimated OR 1.54^22^) and occupational mental stress (estimated OR 1.32^22^) predicted reduced RTW at 2 years. No association with RTW was found for sex (OR 0.22; 95% CI 0.04 to 1.09), BMI (OR 0.90; 95% CI 0.78 to 1.04), general health (OR 1.03; 95% CI 0.98 to 1.08) and physical function (OR 1.06; 95% CI 0.997 to 1.13), smoking status (OR 4.37; 95% CI 0.82 to 23.27)^28^; neither for a combined measure of pain and disability presurgery (estimated OR 1.42^22^), job satisfaction (OR 0.98^26^) and duration of sick leave (OR 1.26^26^). The latter two studies^22 26^ did not present CIs. In patients who underwent revision surgery, surgery with fusion (OR 0.56; 95% CI 0.33 to 0.97), psychiatric comorbidity before revision surgery (OR 0.19; 95% CI 0.05 to 0.68) and opioids use within 1 month of revision surgery (OR 0.44; 95% CI 0.26 to 0.75) predicted reduced RTW at 3 years.^27^

### Quality of evidence

Table 3 shows the quality of evidence for all prognostic factors based on the GRADE criteria. Using these criteria, we assessed whether the quality of the evidence should be downgraded (or upgraded). First, all studies included were explorative studies. Therefore, the starting point for the quality of evidence was moderate. Second, the quality was further downgraded for moderate risk of bias in 16 factors, and third, for imprecision in 19 factors. Factors were only assessed in one study each (ie, one study with the same population and follow-up); therefore, the GRADE item inconsistency was not applicable. We only included studies that investigated prognostic factors of RTW in sciatica populations. Therefore, indirectness, the last item, was never a reason for downgrading. Publication bias was not assessed due to the insufficient number of studies. The quality of evidence of prognostic factors included was graded as either moderate (10 factors), low (six factors) or very low (11 factors). For age, the quality of evidence varied between moderate, low to very low, and for sex between low and very low. This depended on the cohort and time point.

**Table 3:** Quality of the evidence and reasons for downgrading (in bold)

Prognostic factor	Follow-up	Population	Study	Risk of bias	Imprecision	Quality
Demographic factors						
Age	2 years	Mixed	Grøvle *et al* 25	Low	**OR 0.97 (0.93 to 1.00); NS**	Low
Age	4 years	Mixed	Atlas *et al* 23	Low	OR 0.7 (0.6 to 0.8)	Moderate
Age	10 years	Mixed	Atlas *et al* 24	**Moderate**	**OR 0.42 (0.3 to 0.58); CI crosses 0.5**	Very low
Age	3 months	Surgical	Than *et al* 28	**Moderate**	OR 0.92 (0.85 to 0.99)	Low
Female sex	2 years	Mixed	Grøvle *et al* 25	Low	**OR 0.61 (0.31 to 1.22); NS**	Low
Male sex	10 years	Mixed	Atlas *et al* 24	**Moderate**	**OR 0.33 (0.09 to 1.0); NS**	Very low
Male sex	3 months	Surgical	Than *et al* 28	**Moderate**	**OR 0.22 (0.04 to 1.09); NS**	Very low
General health						
General health	2 years	Mixed	Grøvle *et al* 25	Low	OR 1.03 (1.01 to 1.05)	Moderate
General health	6 years	Mixed	Atlas *et al* 23	Low	OR 1.1 (1.0 to 1.2)	Moderate
General health	3 months	Surgical	Than *et al* 28	**Moderate**	**OR 1.03 (0.98 to 1.08); NS**	Very low
Pain and disability						
Low back pain intensity	4 years	Mixed	Atlas *et al* 23	Low	OR 0.8 (0.6 to 0.9)	Moderate
Low back pain frequency	10 years	Mixed	Atlas *et al* 24	**Moderate**	**No data; NS**	Very low
Bothersomeness	2 years	Mixed	Grøvle *et al* 25	Low	OR 0.89 (0.82 to 0.97)	Moderate
Opioid use within 1 month postoperative	3 years	Surgical	O’Donnell *et al* 27	Low	**OR 0.44 (0.26 to 0.75); CI crosses 0.5**	Low
Physical function	10 years	Mixed	Atlas *et al* 24	**Moderate**	OR 1.4 (1.1 to 1.8)	Low
Physical function	3 months	Surgical	Than *et al* 28	**Moderate**	**OR 1.06 (0.997 to 1.13); NS**	Very low
Pain/disability presurgery	2 years	Surgical	Schade *et al* 22	**Moderate**	**No CI presented; NS; <10 participants/PF**	Very low
Psychological factors						
Fear avoidance	2 years	Mixed	Grovle *et al* 25	Low	OR 0.93 (0.90 to 0.97)	Moderate
Fear avoidance*	6 months	Surgical	den Boer *et al* 26	Low	OR 1.09 (1.01 to 1.17)	Moderate
Mental health	10 years	Mixed	Atlas *et al* 24	**Moderate**	**No data; NS**	Very low
Depression*	2 years	Surgical	Schade *et al* 22	**Moderate**	**<10 participants/PF**	Very low
Psychiatric comorbidity	3 years	Surgical	O’Donnell *et al* 27	Low	**OR 0.19 (0.05 to 0.68); CI crosses 0.5**	Low
Occupational mental stress*	2 years	Surgical	Schade *et al* 22	**Moderate**	**<10 participants/PF**	Very low
Passive pain coping*	6 months	Surgical	den Boer *et al* 26	Low	OR 1.08 (1.0 to 1.16)	Moderate
Other health-related factors						
Smoking status	3 months	Surgical	Than *et al* 28	**Moderate**	**OR 4.37 (0.82 to 23.27), NS**	Very low
Clinical examination						
Positive SLR-test	2 years	Mixed	Grøvle *et al* 25	Low	OR 0.44 (0.20 to 0.95)	Moderate
Quebec classification	10 years	Mixed	Atlas *et al* 24	**Moderate**	**No data; NS**	Very low
Care related factors						
Physician expected benefit of surgery	10 years	Mixed	Atlas *et al* 24	**Moderate**	**OR 5.0 (1.65 to 17.7); CI crosses 2.0**	Very low
Initial treatment: surgery	10 years	Mixed	Atlas *et al* 24	**Moderate**	**OR 1.4 (0.46 to 4.6); NS**	Very low
Revision surgery with fusion	3 years	Surgical	O’Donnell *et al* 27	Low	OR 0.56 (0.33 to 0.97)	Moderate
Work-related factors						
Receiving workers’ compensation	4 years	Mixed	Atlas *et al* 23	Low	**OR 0.6 (0.3 to 1.2); NS**	Low
Physical work load*	6 months	Surgical	den Boer *et al* 26	Low	OR 1.19 (1.07 to 1.31)	Moderate
Job satisfaction	6 months	Surgical	den Boer *et al* 26	Low	**No CI presented; NS**	Low
Duration sick leave	6 months	Surgical	den Boer *et al* 26	Low	**No CI presented; NS**	Low

*Prognostic factor for *reduced* return to work; CI, 95% CI; NS, non-significant; PF, prognostic factor.

## Discussion

Work participation is an important goal for sciatica patients of working age. In this study, we reviewed and summarised the prognostic factors of RTW in these patients in both short and long terms, up to 10 years. We found moderate to very low quality GRADE evidence for a wide range of factors to predict RTW: general health, pain and disability, psychological factors, other health-related factors, care and work-related factors. There was insufficient data to observe any trends or differences between factors over time.

Several prognostic factors were also identified in two systematic reviews in non-surgically treated populations with sciatica, though these used recovery,^29^ or pain and disability^30^ as outcomes, as opposed to RTW in the current review. The two sciatica reviews found that physical symptoms like pain intensity and leg pain were prognostic factors, whereas we found that bothersomeness and low back pain predicted reduced RTW. These physical symptoms may be used to identify patients with both an increased risk of reduced recovery and RTW. In contrast, the physical factors better health and functional status predicted RTW, which has been found in low back pain populations as well.^31^ Our study also found that age, sex, job satisfaction and neurological findings showed no association with RTW, confirming earlier findings of no association of these factors with clinical outcomes in sciatica.^29 30^

Psychological factors in prognostic research are useful as these can potentially be modified but can also be used to select patients for specific interventions. Fear of movement is a modifiable psychological factor that predicted reduced RTW in the current review and pain and disability in low back pain patients^31^ and pain at long-term follow-up in sciatica.^32^ Mental stress^29 30^ and passive pain coping^28 30^ were predictors also previously identified. Finally, depression has been shown to predict application for early retirement in sciatica.^32^ These findings underline the conclusion of the North American Spine Society clinical guideline for lumbar disc herniation with radiculopathy that psychosocial variables are important factors that influence recovery.^33^ Screening for these psychological factors may therefore be considered, with subsequent referral to interventions targeting these factors, such as multidisciplinary biopsychosocial rehabilitation.^34^ Psychological therapies, with or without exercise, using a cognitive behavioural approach are also recommended in a recent NICE guideline on low back pain and sciatica to target psychosocial barriers in patients who avoid normal activities by discussing inappropriate beliefs about their condition.^13^

There are some differences between our results and earlier studies. Although two previous reviews^29 30^ showed limited evidence for no association between high work load and poor outcome in terms of pain and disability, we found an association between high work load and reduced RTW in the current study. Considering the difference in outcomes, we hypothesise that work load might be influencing RTW more, as per the current review, than recovery, pain or disability in previous reviews. Also, in our review, a positive SLR test strongly predicted reduced RTW,^26^ and Ashworth *et al* 30 found a positive SLR test to predict ‘worse outcome’ in terms of pain and disability. Verwoerd *et al* reported inconclusive findings with one study finding no association, and another finding a negative association with recovery.^29^ Specificity of the SLR has been found to be limited for diagnostic use, when used in isolation.^35^ Neurological tests, often used in conjunction with SLR testing, did not show that neurological deficits or signs were predictive of RTW in our review.^25 26^ Although the use of SLR testing for diagnostic reasons may be limited, it may serve useful for prognostic reasons for RTW.

RTW rates across the studies included in this review ranged between 67% and 87% and were surprisingly similar, with the exception of the study that assessed RTW after revision surgery.^27^ The RTW rates in the three studies we included for surgically treated patients were 66.9% at 3 months,^28^78% at 6 months^26^ and 81% at 2 years follow-up.^22^ For mixed surgical and conservative populations, the rates were 73% at 2 years,^25^80%–87% at 4 years^22^ and 78% at 10 years follow-up.^24^Patients who underwent revision surgery had lower RTW rates with 40.2% (without fusion) and 27.0% (with fusion).^27^ Apparently, patients undergoing revision surgery represent a different group, with poorer prognostic outcomes, especially when discectomy was combined with fusion. Based on the data presented in the studies included, it is not possible to define to what extent RTW was reduced in patients with unfavourable scores on predictors of RTW, compared with those with favourable scores. To facilitate clinical impact, it is important for future prognostic studies to report separate RTW rates for those with favourable and unfavourable scores on predictors of RTW.

It is suggested that factors influencing recovery may differ between surgically and conservatively treated populations.^30^ In the studies included in this review with mixed populations, the percentage of patients treated surgically were 30%^25^ and 53%.^23^ In the latter, initial treatment did not significantly predict RTW. Most prognostic factors in this review were tested in either mixed or surgical populations, which precludes drawing conclusions on comparability of prognostic factors between these populations. Based on this review and previous reviews,^29 30^ pain intensity seems to be a prognostic factor across all populations, that is, conservative, surgical and mixed, and psychological factors may be important in all patient groups as well.

This study has various strengths and limitations. The data were collected in a systematic way and analysed following current standards for risk of bias assessment, by means of the QUIPS tool,^16^ and grading of the quality of the evidence, by applying the GRADE method.^19^ Most included studies reported imprecise measures. The quality of the prognostic evidence ranged from moderate to very low, meaning that estimates for these latter factors are likely to change when more studies will be available. The results need to be interpreted with caution, as estimates are likely to change when future studies will be available. These future studies should preferably include larger samples of either conservatively or surgically treated patients (or analyse data from these groups separately), and test combinations of factors that have been found to be significant in the current review and previous reviews. These factors are preferably measured with instruments from the core outcome set for low back pain,^36^ and include a standardised instrument to measure RTW, that would allow for meta analyses.^37^

The prognostic evidence from this review, although partially of low to very low quality, may be used to identify potential high risk patients for delayed or no RTW. This information may assist clinicians and occupational healthcare professionals in guiding these high risk patients, in advising or referring them for additional care or vocational rehabilitation, or in managing and counselling patients’ expectations regarding RTW. Monitoring physical and psychological factors also seem relevant as these predict recovery and RTW in the limited number of studies on sciatica in the current review and also in low back pain studies and several clinical guidelines.^13 31^ To enhance work participation, physicians could consider monitoring prognostic factors in patients with sciatica that might benefit from additional clinical management or work-directed care. Given the importance of work participation, we recommend more and well-conducted prognostic studies on this important societal outcome of care.
