# Supplementary material for: Systematic review of prognostic factors for work participation in patients with sciatica
Source: Occup Environ Med. 2019 Jul 11;76(10):772–9. doi: 10.1136/oemed-2019-105797 (PMC6817989; doi:10.1136/oemed-2019-105797)
Supplement: Supplementary file 1 [file oemed-2019-105797supp001.pdf]

Appendix 1 Search strategy in Medline via PubMed

**1 Population, based on NICE guideline**
sciatica[mh] OR radiculopathy[mh] OR radiculitis OR radicular pain* or intervertebral disc displacement[mh] OR (nerve root* AND (pain* or avulsion or compress* or disorder* or pinch* or inflam* or imping* or irritat* or entrap* or trap*))

**2 Ingui filter for prediction studies**
Validat* OR Predict*[ti] OR Rule*) OR (Predict* AND (Outcome* OR Risk OR Model OR Models)) OR ((History OR Variable* OR Criteria OR Scor* OR Characteristic* OR Finding* OR Factor*) AND (Predict* OR Model OR Models OR Decision* OR Identif* OR Prognos*)) OR (Decision* AND (Model OR Models OR Clinical* OR Logistic Models[mh])) OR (Prognostic AND (History OR Variable* OR Criteria OR Scor* OR Characteristic* OR Finding* OR Factor* OR Model OR Models)

**3 Yale filter for prognosis and natural history**
cohort studies[mh] OR prognosis[mh] OR mortality[mh] OR morbidity[mh] OR natural history OR prognost*[tiab] OR course[tiab] OR predict*[tiab] OR outcome assessment[mh] OR outcome*[tiab] OR inception cohort* OR disease progression[mh] OR survival analysis[mh]

**4 Work participation**
Return to Work[mh] OR Employment[mh] OR Occupations[mh] OR Work[mh] OR Workplace[mh] OR Rehabilitation, Vocational[mh] OR Occupational Health[mh] OR Sick Leave[mh] OR Absenteeism[mh] OR Retirement[mh] OR Workers' Compensation[mh] OR return to work[tiab] OR back to work[tiab] OR employment[tiab] OR unemployment[tiab] OR employability[tiab] OR reemployment[tiab] OR work resumption[tiab] OR work status[tiab] OR working age[tiab] OR work re-entry[tiab] OR sick leave[tiab] OR vocational rehabilitation[tiab] OR job[tiab] OR work capacity[tiab] OR productivity[tiab]

**5 1 AND (2 OR 3) AND 4**

Appendix 2 Excluded studies after full text assessment

1 Abásolo L, Lajas C, León L, Carmona L, Macarrón P, Candelas G, Blanco M, Jover JA. Prognostic factors for long-term work disability due to musculoskeletal disorders. Rheumatol Int. 2012 Dec;32(12):3831-9.

2 Adogwa O, Huang MI, Thompson PM, Darlington T, Cheng JS, Gokaslan ZL, Gottfried ON, Bagley CA, Anderson GD, Isaacs RE. No difference in postoperative complications, pain, and functional outcomes up to 2 years after incidental durotomy in lumbar spinal fusion: a prospective, multi-institutional, propensity-matched analysis of 1,741 patients. Spine J. 2014 Sep 1;14(9):1828-34.

3 Ahn SS, Kim SH, Kim DW, Lee BH. Comparison of Outcomes of Percutaneous Endoscopic Lumbar Discectomy and Open Lumbar Microdiscectomy for Young Adults: A Retrospective Matched Cohort Study. World Neurosurg. 2016 Feb;86:250-258

4 Almeida DB, Poletto PH, Milano JB, Leal AG, Ramina R. Is preoperative occupation related to long-term pain in patients operated for lumbar disc herniation? Arq Neuropsiquiatr. 2007 Sep;65(3B):758-63.

5 Andersson GB, Svensson HO, Odén A. The intensity of work recovery in low back pain. Spine. 1983 Nov-Dec;8(8):880-4.

6 Asher AL, Devin CJ, Archer KR, Chotai S, Parker SL, Bydon M, Nian H, Harrell FE, Speroff T, Dittus RS, Philips SE, Shaffrey CI, Foley KT, McGirt MJ. An analysis from the Quality Outcomes Database, Part 2. Predictive model for return to work after elective surgery for lumbar degenerative disease. J Neurosurg.: Spine. 2017 Oct;27(4):370-381

7 Atlas SJ, Tosteson TD, Blood EA, Skinner JS, Pransky GS, Weinstein JN. The impact of workers' compensation on outcomes of surgical and nonoperative therapy for patients with a lumbar disc herniation: SPORT. Spine. 2010 Jan 1;35(1):89-97.

8 Atlas SJ, Tosteson TD, Hanscom B, Blood EA, Pransky GS, Abdu WA, Andersson GB, Weinstein JN. What is different about workers' compensation patients? Socioeconomic predictors of baseline disability status among patients with lumbar radiculopathy. Spine. 2007 Aug 15;32(18):2019-26.

9 Balagué F, Nordin M, Sheikhzadeh A, Echegoyen AC, Brisby H, Hoogewoud HM, Fredman P, Skovron ML. Recovery of severe sciatica. Spine. 1999 Dec 1;24(23):2516-24.

10 Barrios C, Ahmed M, Arrotegui JI, Björnsson A. Clinical factors predicting outcome after surgery for herniated lumbar disc: an epidemiological multivariate analysis. J Spinal Disord. 1990 Sep;3(3):205-9.

11 Bederman SS. Predicting prognosis in sick-listed low back pain patients: sneaking a peak inside the black box. Spine J. 2010 Aug;10(8):728-30.

12 Bejia I, Younes M, Zrour S, Touzi M, Bergaoui N. Factors predicting outcomes of mechanical sciatica: a review of 1092 cases. Joint Bone Spine. 2004 Nov;71(6):567-71.

13 Boshuizen HC, Hulshof CT, Bongers PM. Long-term sick leave and disability pensioning due to back disorders of tractor drivers exposed to whole-body vibration. Int Arch Occup Environ Health. 1990;62(2):117-22.

14 Bovenzi M, Schust M, Menzel G, Hofmann J, Hinz B. A cohort study of sciatic pain and measures of internal spinal load in professional drivers. Ergonomics. 2015;58(7):1088-102

15 Briseño MR, Phukan RD, Leonard DA, Herzog TL, Cho CH, Schwab JH, Wood KB, Bono CM, Cha TD. The influence of adjacent level disc disease on discectomy outcomes. Eur Spine J. 2016 Jan;25(1):230-4.

16 Carragee EJ. Psychological screening in the surgical treatment of lumbar disc herniation. Clin J Pain. 2001 Sep;17(3):215-9.

17 Chen HN, Tsai YF. A predictive model for disability in patients with lumbar disc herniation. J Orthop Sci. 2013 Mar;18(2):220-9.

18 den Boer JJ, Oostendorp RA, Beems T, Munneke M, Evers AW. Continued disability and pain after lumbar disc surgery: the role of cognitive-behavioral factors. Pain. 2006 Jul;123(1-2):45-52.

19 Dewing CB, Provencher MT, Riffenburgh RH, Kerr S, Manos RE. The outcomes of lumbar microdiscectomy in a young, active population: correlation by herniation type and level. Spine. 2008 Jan 1;33(1):33-8.

20 Donceel P, Du Bois M. Fitness for work after surgery for lumbar disc herniation: a retrospective study. Eur Spine J (1998) 7 :29–35.

21 Donceel P, Du Bois M. Predictors for work incapacity continuing after disc surgery. Scand J Work Environ Health 1999;25(3):264-271.

22 Dubourg G, Rozenberg S, Fautrel B, Valls-Bellec I, Bissery A, Lang T, Faillot T, Duplan B, Briançon D, Levy-Weil F, Morlock G, Crouzet J, Gatfosse M, Bonnet C, Houvenagel E, Hary S, Brocq O, Poiraudeau S, Beaudreuil J, de Sauverzac C, Durieux S, Levade MH, Esposito P, Maitrot D, Goupille P, Valat JP, Bourgeois P. A pilot study on the recovery from paresis after lumbar disc herniation. Spine. 2002 Jul 1;27(13):1426-31.

23 Findlay GF, Hall BI, Musa BS, Oliveira MD, Fear SC. A 10-year follow-up of the outcome of lumbar microdiscectomy. Spine. 1998 May 15;23(10):1168-71.

24 Furunes H, Hellum C, Brox JI, Rossvoll ., Espeland A, Berg L, Brogger HM, Smastuen MC, Storheim K. Lumbar total disc replacement: predictors for long-term outcome. Eur Spine J. 2017 Nov;1-10

25 Grøvle L, Haugen AJ, Natvig B, Brox JI, Grotle M. The prognosis of self-reported paresthesia and weakness in disc-related sciatica. Eur Spine J. 2013 Nov;22(11):2488-95.

26 Guan J, Bisson EF, Dailey AT, Hood RS, Schmidt MH. Comparison of clinical outcomes in the national neurosurgery quality and outcomes database for open versus minimally invasive transforaminal lumbar interbody fusion. Spine. 2016;41(7):E416-E421

27 Habbema JD, Braakman R, Blaauw G, Slebus FG, Singh R. Patients' condition one year following surgery for a lumbosacral radicular syndrome. Ned Tijdschr Geneeskd. 1989 Dec 30;133(52):2615-9.

28 Hägg O, Fritzell P, Ekselius L, Nordwall A; Swedish Lumbar Spine Study. Predictors of outcome in fusion surgery for chronic low back pain. A report from the Swedish Lumbar Spine Study. Eur Spine J. 2003 Feb;12(1):22-33.

29 Hakkinen A, Ylinen J, Kautiainen H, Airaksinen O, Herno A, Kiviranta I. Does the outcome 2 months after lumbar disc surgery predict the outcome 12 months later? Disabil Rehabil. 2003 Sep 2;25(17):968-72.

30 Harris IA, Dantanarayana N, Naylor JM. Spine surgery outcomes in a workers' compensation cohort. ANZ J Surg. 2012 Sep;82(9):625-9.

31 Heymans MW, Ford JJ, McMeeken JM, Chan A, de Vet HC, van Mechelen W. Exploring the contribution of patient-reported and clinician based variables for the prediction of low back work status. J Occup Rehabil. 2007 Sep;17(3):383-97.

32 Hider SL, Whitehurst DG, Thomas E, Foster NE. Pain location matters: the impact of leg pain on health care use, work disability and quality of life in patients with low back pain. Eur Spine J. 2015 Mar;24(3):444-51.

33 Hodges SD, Humphreys SC, Eck JC, Covington LA, Harrom H. Predicting factors of successful recovery from lumbar spine surgery among workers' compensation patients. J Am Osteopath Assoc. 2001 Feb;101(2):78-83.

34 Hordijk JC, Luijsterburg PAJ, Koes BW, Verhagen AP. Prognostische factoren bij een lumbosacraal radiculair syndroom. Huisarts Wet 2010;53(9):474-8.

35 Jensen OK, Nielsen CV, Stengaard-Pedersen K. One-year prognosis in sick-listed low back pain patients with and without radiculopathy. Prognostic factors influencing pain and disability. Spine J. 2010 Aug;10(8):659-75.

36 Jensen OK, Stengaard-Pedersen K, Jensen C, Nielsen CV. Prediction model for unsuccessful return to work after hospital-based intervention in low back pain patients. BMC Musculoskelet Disord. 2013 Apr 19;14:140.

37 Jensen LD, Frost P, Schiøttz-Christensen B, Maribo T, Christensen MV, Svendsen SW. Predictors of vocational prognosis after herniated lumbar disc: a two-year follow-up study of 2039 patients diagnosed at hospital. Spine. 2011 May 20;36(12):E791-7.

38 Johansson AC, Linton SJ, Rosenblad A, Bergkvist L, Nilsson O. A prospective study of cognitive behavioural factors as predictors of pain, disability and quality of life one year after lumbar disc surgery. Disabil Rehabil. 2010;32(7):521-9.

39 Kääriä S, Laaksonen M, Leino-Arjas P, Saastamoinen P, Lahelma E. Low back pain and neck pain as predictors of sickness absence among municipal employees. Scand J Public Health. 2012 Mar;40(2):150-6

40 Kerr D, Zhao W, Lurie JD. What Are Long-term Predictors of Outcomes for Lumbar Disc Herniation? A Randomized and Observational Study. Clin Orthop Relat Res. 2015 Jun;473(6):1920-30.

41 Lønne G, Solberg TK, Sjaavik K, Nygaard ØP. Recovery of muscle strength after microdiscectomy for lumbar disc herniation: a prospective cohort study with 1-year follow-up. Eur Spine J. 2012 Apr;21(4):655-9.

42 Lurie JD, TostesonTD,  Tosteson ANA, Zhao W, Morgan TS, Abdu WA, Herkowitz H, Weinstein JN. Surgical versus Non-Operative Treatment for Lumbar Disc Herniation: Eight-Year Results for the Spine Patient Outcomes Research Trial (SPORT). Spine. 2014;39(1):3-16

43 Lv Y, Chen J, Wu Y, Chen X, Liu Y, Chu Z, Sheng L, Qin R, Chen M.Three-year postoperative outcomes between MIS and conventional TLIF in1-segment lumbar disc herniation. Minim Invasive Ther Allied Technol. 2017 May; 26(3):168-176

44 McGirt MJ, Parker SL, Mummaneni P, Knightly J, Pfortmiller D, Foley K, Asher AL. Is the use of minimally invasive fusion technologies associated with improved outcomes after elective interbody lumbar fusion? Analysis of a nationwide prospective patient-reported outcomes registry. Spine J. 2017 Jul;17(7):922-932

45 McGirt MJ, Bydon M, Archer KR, Devin CJ, Chotai S, Parker SL, Nian H, Harrell FE, Speroff T, Dittus RS, Philips SE, Shaffrey CI, Foley KT, Asher AL. An analysis from the Quality Outcomes Database, Part 1. Disability, quality of life, and pain outcomes following lumbar spine surgery: Predicting likely individual patient outcomes for shared decision-making. J Neurosurg: Spine. 2019 Oct;27 (4):357-369

46 Nygaard OP, Romner B, Trumpy JH. Duration of symptoms as a predictor of outcome after lumbar disc surgery. Acta Neurochir (Wien). 1994;128(1-4):53-6.

47 Nygaard OP, Kloster R, Solberg T. Duration of leg pain as a predictor of outcome after surgery for lumbar disc herniation: a prospective cohort study with 1-year follow up. J Neurosurg. 2000 Apr;92(2 Suppl):131-4.

48 Nykvist F, Hurme M, Alaranta H, Kaitsaari M. Severe sciatica: a 13-year follow-up of 342 patients. Eur Spine J. 1995;4(6):335-8.

49 Nykvist F, Hurme M, Alaranta H, Miettinen ML. Social factors and outcome in a five-year follow-up study of 276 patients with sciatica. Scand J Rehabil Med. 1991;23(1):19-26.

50 Peul WC, Brand R, Thomeer RT, Koes BW. Influence of gender and other prognostic factors on outcome of sciatica. Pain. 2008 Aug 15;138(1):180-91

51 Puolakka K,  Ylinen J,  Neva MH, Kautiainen K,  Häkkinen A. Risk factors for back pain-related loss of working time after surgery for lumbar disc herniation: a 5-year follow-up study. Eur Spine J. 2008;17:386–392

52 Rasmussen C. Lumbar disc herniation: social and demographic factors determining duration of disease. Eur Spine J. 1996;5(4):225-8.

53 Rompe JD, Eysel P, Zöllner J, Heine J. Prognostic criteria for work resumption after standard lumbar discectomy. Eur Spine J. 1999;8(2):132-7.

54 Rönnberg K, Lind B, Zoëga B, Halldin K, Gellerstedt M, Brisby H. Patients' satisfaction with provided care/information and expectations on clinical outcome after lumbar disc herniation surgery. Spine. 2007 Jan 15;32(2):256-61.

55 Triebel J, Snellman G, Sanden B, Stromqvist F, Robinson Y. Women do not fare worse than men after lumbar fusion surgery: Two-year follow-up results from 4,780 prospectively collected patients in the Swedish National Spine Register with lumbar degenerative disc disease and chronic low back pain. Spine J. 2017 May;17(5):656-662

56 Tu Z., Li YW, Wang B, Lu G, Li L, Kuang L, Dai Y. Clinical Outcome of Full-endoscopic Interlaminar Discectomy for Single-level Lumbar Disc Herniation: A Minimum of 5-year Follow-up Pain Physician. 2017 Mar;20(3):E425-E430

57 Zhang Y, Yang XJ, Zeng TH, Qiu YY, Wang YT, Liang FG. A retrospective study of epidural and intravenous steroids after percutaneous endoscopic lumbar discectomy for large lumbar disc herniation. Chin J Traumatol. 2017 Feb;20(1):34-38

58 Zieger M, Luppa M, Meisel HJ, Günther L, Winkler D, Toussaint R, Stengler K, Angermeyer MC, König HH, Riedel-Heller SG. The impact of psychiatric comorbidity on the return to work in patients undergoing herniated disc surgery. J Occup Rehabil. 2011 Mar;21(1):54-65.
